# Intraocular Lens power calculation after laser refractive surgery: A Meta-Analysis

**DOI:** 10.1038/s41598-020-59487-1

**Published:** 2020-02-14

**Authors:** Hui Chen, Xinyi Chen, Hanle Wang, Zhi Fang, Ke Yao

**Affiliations:** 1 Eye Center, Second Affiliated Hospital, School of Medicine, Zhejiang University, Hangzhou Zhejiang, P.R. China; 2 Key Laboratory of Ophthalmology of Zhejiang Province, Hangzhou Zhejiang, P.R. China; 3 The Eye Hospital, Wenzhou Medical University, Wenzhou Zhejiang, P.R. China

**Keywords:** Corneal diseases, Lens diseases

## Abstract

There are an increasing number of people who have had refractive surgery now developing cataract. To compare the accuracy of different intraocular lens (IOL) power calculation formulas after laser refractive surgery (photorefractive keratectomy or laser *in situ* keratomileusis), a comprehensive literature search of PubMed and EMBASE was conducted to identify comparative cohort studies and case series comparing different formulas: Haigis-L, Shammas-PL, SRK/T, Holladay 1 and Hoffer Q. Seven cohort studies and three observational studies including 260 eyes were identified. There were significant differences when Hoffer Q formula compared with SRK/T, Holladay 1. Holladay 1 formula produced less prediction error than SRK/T formula in double-K method. Hoffer Q formula performed best among SRK/T and Holladay 1 formulas in total and single-K method. In eyes with previous data, it is recommended to choose double-K formula except SRK/T formula. In eyes with no previous data, Haigis-L formula is recommended if available, if the fourth formula is unavailable, single-k Hoffer Q is a good choice.

## Introduction

According to the latest assessment, cataracts account for 51% of the world’s blindness, that is about 20 million people. Thus, cataract is still the main cause of blindness^1^. It only can be removed by surgery. In the past few decades, the surgical technique has gone earth-shaking changes, from ICCE, ECCE to phacoemulsification and femtosecond laser-assisted cataract surgery^2^. With the development of intraocular lenses (IOL), from unfoldable IOL to foldable IOL, until now there are a number of functional IOLs such as Toric IOL, multifocal IOL, Symfony IOL and adjustable IOL, resulting in more precise and comfortable postoperative refractive outcomes^2^, symbolize cataract surgery coming into refractive times. Thus, patients hope not only clear, comfortable but also up to ideal refractive status.

However, lens calculations are not perfect. Residual postoperative refractive error can be common, especially in patients who undergone refractive corneal surgery before and are now developing cataracts^3–6^. In spite of a good many of methods for IOL power calculation, the postoperative refractive errors are unpredictable in these patients compared to those who have no refractive surgery experience. There are three kinds of reasons responsible for the prediction error in intraocular lens calculation after refractive surgery: instrument error, refractive index error and formula error^7^.

The first-generation formula is derived from the principle of geometric optics and using the thin lens imaging formula, represented by the Binkhorst^8^, Colenbrander and regression formula SRK I formula^9^. The second-generation formula are the regression formula SRKII formula that introduced after the improvement of SRK I formula and the Binkhorst II formula based on the correction of the axial axis and the anterior chamber depth (ACD)^10^. Soon after, the third-generation formulas came out. Holladay *et al*.^11^ introduced the corneal curvature into the ACD calculation formula. Retzlaf *et al*.^12^ based on the SRK II formula, summed up the analysis of 1677 cases of the eye and came up with theoretical formula SRK/T. There is also the Hoffer Q formula, relying on a personalized ACD, corneal curvature, and axial length^13^. The fourth-generation formulas include Holladay II formula and the Haigis formula^14^. Compared with the previous three generations of formulas, the fourth-generation formula takes the effective IOL position (ELP) into account, and to some extent realizes the individualization of the IOL calculation. Recently, the fifth-generation formula Barrett Universal II has been applied to the clinic^15^.

Refractive surgery is increasingly accepted and welcomed by the public. Over the past couple of years, refractive surgery technique has been developing rapidly, including radial keratotomy (RK), photorefractive keratectomy (PRK), laser *in situ* keratomileusis (LASIK) and small-incision lenticule extraction (SMILE) etc. There is no any formula has been universally acknowledged as having high accuracy in various eyes^16^ and the eye features after refractive surgery are more complicated. Utilizing the erroneous K-reading in post-operative eyes into standard IOL power calculation formulas leads to overestimation of keratometry diopters, then resulting in underestimated power of IOL for hyperopia after cataract surgery^17–19^. Patients undergoing refractive surgery usually have higher requirements for vision. Thus, they also hope to get perfect visual acuity after cataract surgery, so the surgeon needs to find proper formula to ensure the accuracy of the intraocular lens power calculation. For this subset of patients, a meta-analysis comparing different formulas to calculate IOL power was performed.

## Results

### Literature search

**Figure 1:**
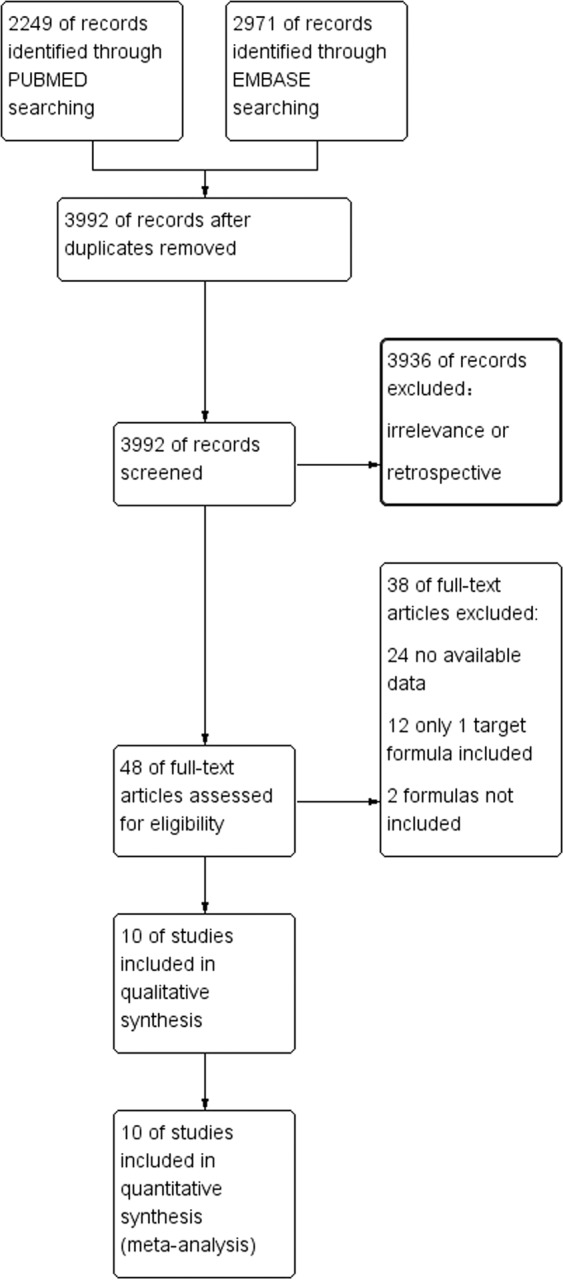
Flow diagram of the literature search in this meta-analysis.

The flowchart (Fig. 1) shows the literature search process. After removing the duplicates, there are 3,992 articles in total. Among them, 3936 records were excluded because of irrelevance or retrospective research. 48 articles were read full text and then assessed. 22 of them had the irrelevant data to our outcomes of interest, 12 of them were excluded because of only one target IOL calculation formula and the formulas in 2 studies were not included. Finally, 10 articles^3,19–27^ meeting all of the screening criteria were included in this meta-analysis.

### Characteristics of included studies

In the present meta-analysis, ten studies were included, seven of which were comparative cohort studies and three of which were prospective case series. Table 1 shows the characteristics of the ten studies. The quality assessment(NOS scale) of the comparative cohort studies and case series studies is shown in Table 1. Overall, 267 eyes having refractive surgery history were analyzed. The mean age of the patients in these included studies ranged from 28 to 61 years and the mean axial length(AL) ranged from 25 to 30 mm. Six studies were completed in America, one in China, one in Egypt, and the remaining two in Europe. The follow-up duration ranged from 1 month to 2 years.

**Table 1:** Characteristics of included studies.

Study ID	Country	Study design	Mean age	No. of eyes	Surgery	AL (mm)	Formula						ME	MAE	Within percentage(D)	Follow-up
							Haigis-L	Shammas-PL formula	Holladay 1	SRK/T	Hoffer Q	Holladay 2				
Wu 2017^20^	China	Prospective cohort	50.3 ± 9.0	10	phaco	30.06 ± 2.87	✓	✓					✓	NA	±0.5,1.0,1.5	3 months
Helaly 2016^19^	Egypt	Prospective cohort	51.27 ± 7.31	45	phaco	28.66 ± 2.78	✓	✓		Double-K			✓	✓	±0.5,1.0,2.0	4 months
Huang 2013^21^	America	Prospective cohort	61.5 ± 8.0	46	phaco	NA	✓	✓					✓	NA	±0.5,1.0	1 month
Savini 2010^22^	America	Prospective cohort	52.5 ± 9.6	28	phaco	27.84 ± 1.90			Double-K	Double-K			✓	✓	NA	1 month
Jin 2010^23^	Germany	Prospective cohort	31.81 ± 7.49	28	phaco	24.94 ± 1.08	✓			✓	✓		NA	✓	NA	1 year
Arce 2009^24^	America	Prospective cohort	NA	32	phaco	NA	✓		✓	✓	✓		✓	✓	NA	2 years
Shammas 2007^3^	America	Prospective cohort	28 to 67	15	phaco	27.19 ± 2.52	✓	✓	Double-K	Double-K	Double-K	Double-K	✓	✓	±1.0,	12 weeks
Savini 2018^25^	Italy	Prospective case series	56.4 ± 8.3	22	phaco	26.7 ± 1.7			Double-K	Double-K			✓	NA	±0.5,0.75,1.0	NA
Savini 2015^26^	America	Prospective case series	50.1 ± 9.2	30	phaco	27.06 ± 2.05			Double-K	Double-K	Double-K		✓	NA	±0.5	NA
Wang 2004^27^	America	Prospective case series	50	11	phaco	25.99			✓	✓	✓	✓	✓	✓	NA	1 year

AL, axial length; ME, mean error; MAE, mean absolute error; NA, not available.

### Outcomes

#### Haigis-L vs Shammas-PL

**Figure 2:**
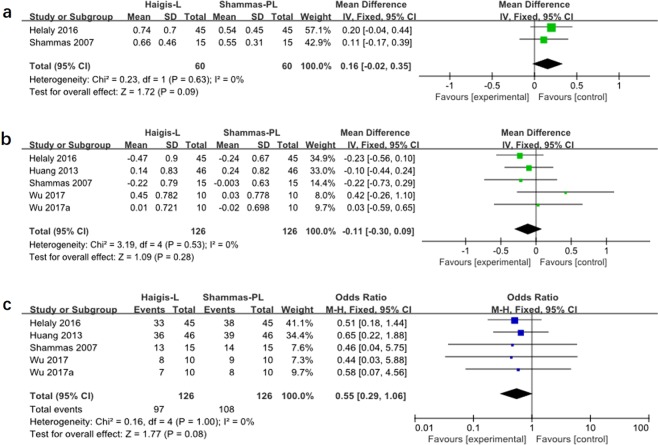
Forest plots comparing the MAE(**a**), ME(**b**) between Haigis-L and Shammas-PL. and the percentage of eyes within ±1.00D of prediction error between Haigis-L and Shammas-PL (**c**).

There were four studies comparing Haigis-L and Shammas-PL formulas, two of which reported MAE(Fig. 2a) and all four studies reported ME(Fig. 2b). There was no significant difference both in MAE (WMD: 0.16, 95% CI: −0.02 to 0.35, P = 0.09) and ME (WMD: −0.11, 95% CI: −0.30 to 0.09, P = 0.53) between Haigis-L and Shammas-PL formulas. Similarly, the percentage of eyes within ±1.00D of prediction error showed no significant difference between these two formulas.(Fig. 2c)

#### Haigis-L vs SRK/T

**Figure 3:**
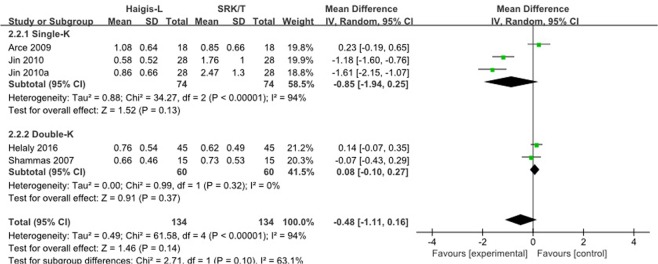
Forest plots comparing the MAE between Haigis-L and SRK/T.

There were four studies reporting Haigis-L and SRK/T formulas in MAE, showing no significant difference between two formulas in total. Then two subgroups (Single-K and Double-K) were added to analyze as shown in Fig. 3. Neither single-K group nor double-K group had significant difference between two formulas.

#### Haigis-L vs Hoffer Q

**Figure 4:**
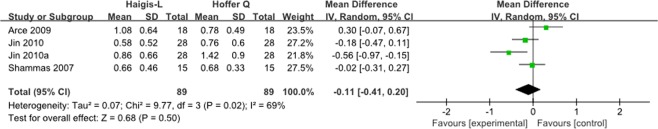
Forest plots comparing the MAE between Haigis-L and Hoffer Q.

Three studies compare Haigis-L and Hoffer Q formulas. There was no significant difference in MAE (WMD: −0.11, 95% CI: −0.41 to 0.20, P = 0.50) (Fig. 4). Similar outcome in ME was shown in Fig. S1.

#### Haigis-L vs Holladay 1

**Figure 5:**
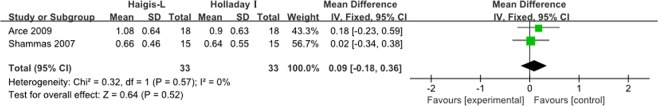
Forest plots comparing the MAE between Haigis-L and Holladay 1.

No significant difference was found in MAE(WMD: 0.09, 95% CI: −0.18 to 0.36, P = 0.52) and ME(WMD: −0.62, 95% CI: −1.45 to 0.21, P = 0.14)when comparing Haigis-L and Holladya1 formulas of two articles, as shown in Figs. 5 and S2 respectively.

#### SRK/T vs Holladay 1

**Figure 6:**
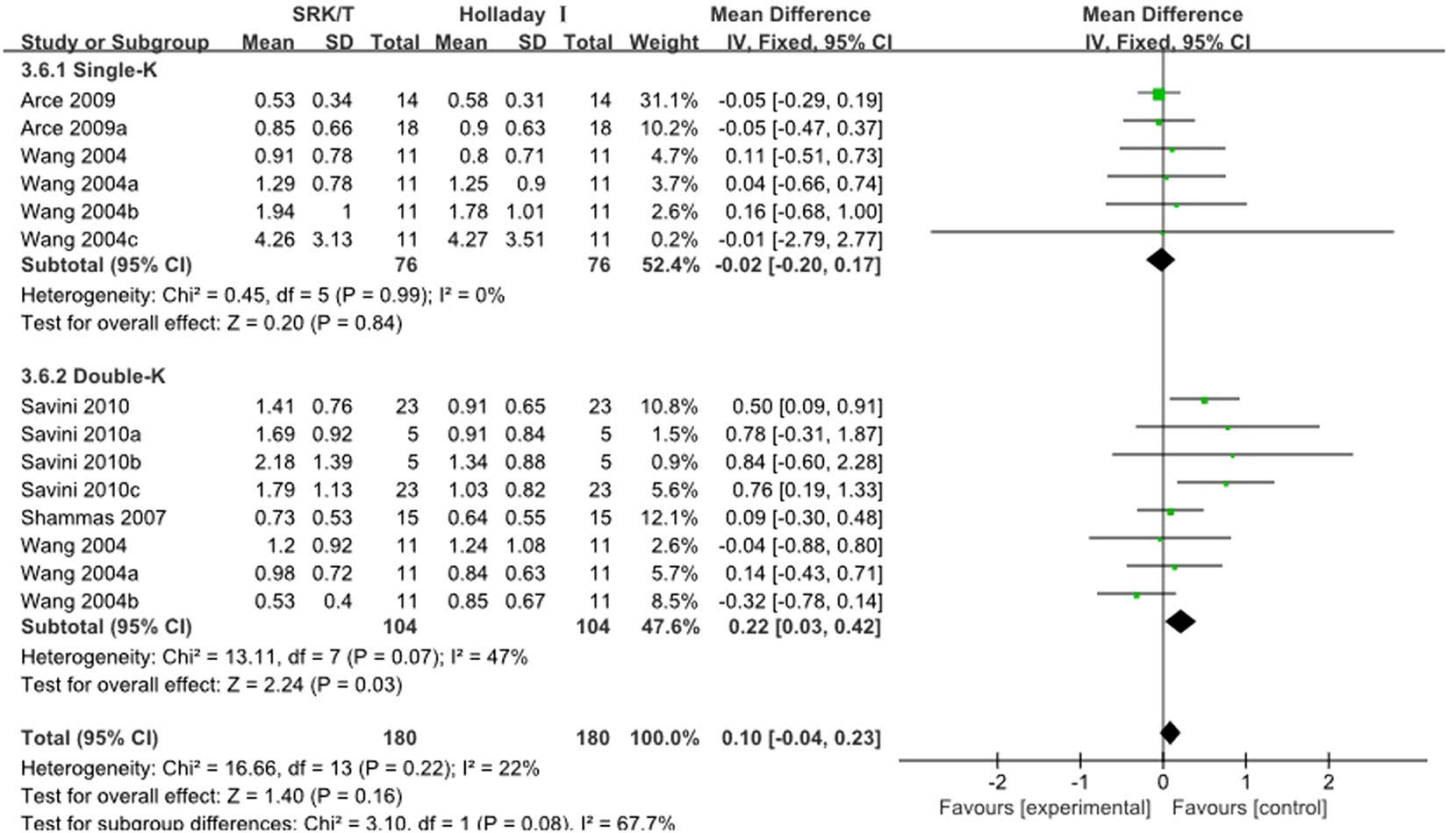
Forest plots comparing the MAE between SRK/T and Holladay 1.

There were six studies comparing SRK/T and Holladay 1 formulas, four of which reported MAE(Fig. 6) and all six studies reported ME(Fig. S3). The forest plot of the comparison in Fig. 6 showed no significant difference between SRK/T and Holladay1 formulas in the MAE in total. Then two subgroups (Single-K and Double-K) were considered to estimate. The MAE in the double-K subgroup of Holladay1 was significantly lower than that of SRK/T(WMD: 0.22, 95% CI: 0.03 to 0.42, P = 0.03) while there was no significant difference in the single-k subgroup. As for ME, no significant difference was found.

#### SRK/T vs Hoffer Q

**Figure 7:**
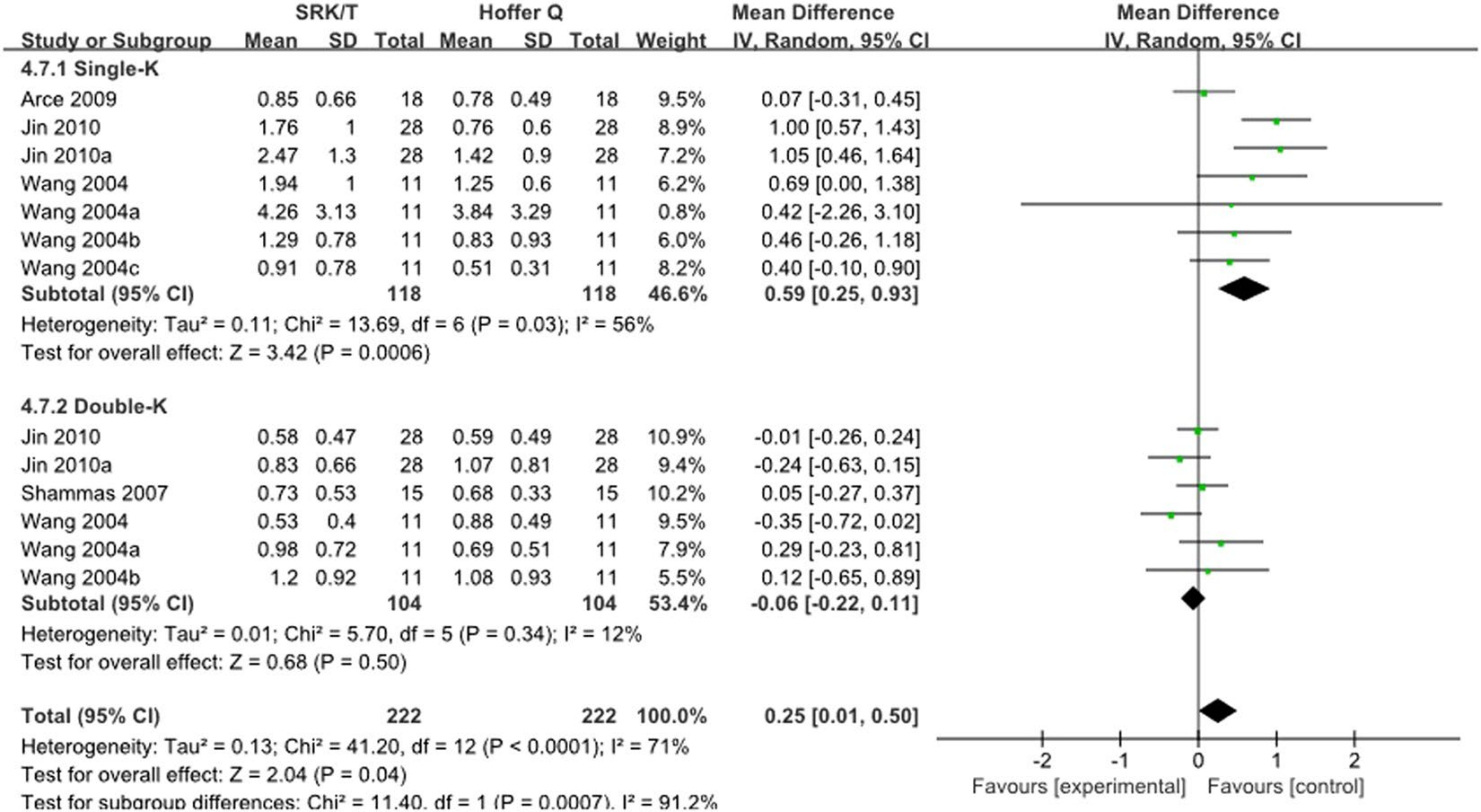
Forest plots comparing the MAE between SRK/T and Hoffer Q.

The MAE was compared between SRK/T and Hoffer Q formulas in four studies. As shown in Fig. 7, the MAE of Hoffer Q was significantly lower than that of SRK/T in total(WMD: 0.26, 95% CI: 0.03 to 0.50, P = 0.03). Two subgroups (Single-K and Double-K) were added to access MAE of SRK/T and Hoffer Q formulas. It was significantly different between them in the single-K group (WMD: 0.59, 95% CI: 0.25 to 0.93, P = 0.0006) based on a random-effects model. However, no significant difference in the double-K group was found.

#### Holladay 1 vs Hoffer Q

**Figure 8:**
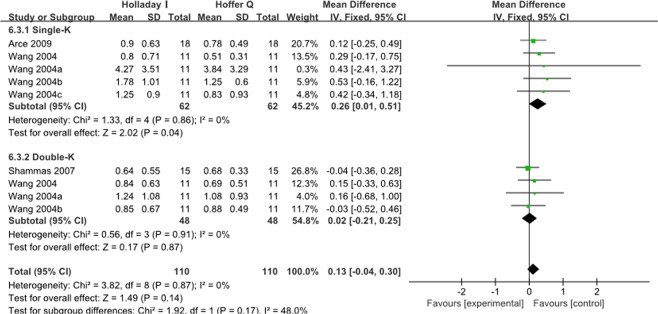
Forest plots comparing the MAE between Holladay 1 and Hoffer Q.

As shown in Fig. 8, the MAE of Hoffer Q was significantly lower than that of Holladay 1 in single-K group(WMD: 0.26, 95% CI: 0.01 to 0.51, P = 0.04). And there was no significant difference between these two formulas in double-K group and in total. Similarly, the ME of Hoffer Q was significantly lower than that of Holladay 1 in single-K group (WMD: 0.26, 95% CI: 0.01 to 0.51, P = 0.04) (Fig. S4).

### Heterogeneity and publication bias

Some of the outcomes displayed great heterogeneity and then used random‐effect model. The sensitivity analysis showed that I^2^ significantly decreased by omitting Jin 2010 in the comparison between Haigis-L and SRK/T and between SRK/T and Hoffer Q.

## Discussion

The results of the present meta-analysis demonstrated that Holladay1 formula produced less prediction error than SRK/T formula in double-K method. Hoffer Q formula performed best among SRK/T and Holladay1 formulas in single-K method. While there was no significant difference between double-K Hoffer Q and double-K SRK/T formulas or double-K Hoffer Q and double-K Holladay1 formulas. The MAE was no significant difference when Haigis-L formula compared with Shammas-PL, Hoffer Q, SRK/T or Holladay 1 formulas. While the ME of Haigis-L formula performed better than Hoffer Q formula.

Aramberri^17^ introduced that there are two main challenges in intraocular lens power calculation after refractive surgery: inaccurate estimation of corneal power and inaccurate calculation formula. In clinic, current topography can only measure anterior corneal power. It is inaccurate to measure the net corneal power in eyes undergone keratorefractive surgery. Because the relationship between the anterior and posterior corneal radius of curvature has been changed, it means that it is no longer 7.5/6.3. This will make the values of different corneal refractive indices meaningless (standardized index of refraction = 1.3375; SRK/T = 1.3333).

In addition, K value is applied in 2 ways in third-generation IOL calculation formulas: (1) to compute the effective lens position (ELP) and (2) to calculate the IOL power^28^. The first step is considered to estimate the anterior chamber depth (ACD). That means assuming the ACD is constant after refractive surgery, then using a lower than original K-value due to keratorefractive surgery will lead to an underestimation of the ELP and then an underestimation of IOL power, eventually a postoperative hyperopia drift. To solve this problem, Aramberri^17^ proposed the “double-K method”, in which K value before operation was used to calculate the ELP, and K value after surgery was used to calculate IOL power. It made it possible to obtain more accurate IOL power. In the present meta-analysis, for single formula, mean value of double-k method was lower than single-k method. There was no significant difference between Hoffer Q and SRK/T, Hoffer Q and Holladay 1 in double-k method. To a certain extent, actually, we believed accuracy of above formulas was improved in double-k method so that obvious difference was cannot be detected. However, the MAE of Hoffer Q was significantly lower than SRK/T or Holladay 1 in single-k method.

Haigis-L formula is commonly applied to calculate IOL power in the clinic. Together with Shammas-PL formula^3^ are belong to no-history method. Unlike most formulas, Shammas-PL and Haigis-L can determine ELP without knowing the central corneal refraction. The results of the present study are consistent with previous studies. Chen *et al*.^29^ found it was similarly accurate when the Shammas-PL formula was compared with the Haigis-L method in eyes after laser refractive surgery. And it was consistent with our result. While Saiki *et al*.^30^ reported that Shammas-PL performed better than Haigis-L formula due to relatively few calculation parameters and fewer measurement errors. In the present study, additionally, the MAE of Haigis-L formula was not significantly different with Hoffer Q, SRK/T or Holladay 1 formulas. The comparison between Shammas-PL and the third-generation formulas did not be analyzed because of too little study. Recent years, Barrett True-K formula has been proposed for IOL power calculation in post- keratorefractive surgery. The refractive prediction error of the Barrett True-K formula is comparable to that of Haigis-L or Shammas formula, and even better than that of Haigis-L or Shammas formula^31,32^. Future more studies are needed to explore it.

Unavoidably, there are several limitations in this meta‐analysis. First, only a small set of researches were enrolled in this meta-analysis, resulting in some comparison available in only 1 combination. Second, the anterior corneal surfaces in studies were obtained by different instruments (Scheimpflug imaging, Partial coherence interferometry, PCI, Optical coherence tomography) rather than the same topography device. We believe that the instruments used in different hospitals are not the same, which is more in line with the actual situation.

To conclusion, the methods fall into two categories: clinical history and no history. Double-k formulas are recommended for IOL power calculation in eyes with pre-keratorefractive surgery data due to their improved accuracy, while double-k SRK/T is not recommended. Haigis-L formula, if available, is recommended in eyes with no history data. Single-K Hoffer Q formula would be a good choice if there is no fourth-generation formula. Haigis-L is not significantly different with Shammas-PL formula, thus it is no need to introduce Shammas-PL formula into instruments, after all, Haigis-L formula has been widely used.

## Methods

### Literature search

PubMed and EMBASE were searched for articles dated up to March 2019, using the following terms: (PRK OR LASIK) AND (cataract OR IOL OR intraocular lens). There is no restriction on the language of the publication. Two independent reviewers (H.C and XY.C) first conducted a preliminary review of titles and abstracts, and then analyzed the full articles to select the studies that met our predefined criteria. Disagreement between two reviewers was resolved through careful discussion—involving a third reviewer when necessary—until a consensus was reached.

### Inclusion and exclusion criteria

Included articles met the following inclusion criteria: (1) focused on individuals with corneal refractive surgery history; (2) eyes undergone uncomplicated cataract surgery with in‐the‐bag fixated IOL implantation; (3) used at least two of the selected IOL power calculation formulas (Haigis-L, Shammas-post LASIK, SRK/T, Holladay 1, Hoffer Q). Articles were excluded when they: (1) eyes with other disorders e.g., glaucoma, uveitis, or macular degeneration; (2) using toric, multifocal or piggyback IOL; (3) were review articles or discussion papers, conference abstracts, retrospective studies or studies done on animals.

### Quality assessment

Two reviewers evaluated the quality assessment of the cohort study and case series independently by the Newcastle-Ottawa Scale (NOS)^33^. This scale uses a total of nine stars: four in patient selection, two in comparability, and three in outcome assessment. A score ≥6 indicates good quality.

### Data extraction

A standard form was used to extract the data, including authors, country and year of publication, study design, numbers, age and sex of patients, eye sample size, left-right eye proportion, mean absolute errors (MAE), mean arithmetic error(ME) and the percentage of eyes within ±1.00D of prediction error data. A second researchers double-checked all data.

### Statistical analysis

Using RevMan software (version 5.3; Cochrane Collaboration, Oxford, United Kingdom) to perform all statistical analyses. The weighted mean difference (WMD) with a 95% CI was calculated for the continuous outcomes. A p-value less than 0.05 was considered statistically significant. The odds ratio (OR) was calculated to estimate the percentage of eyes within ± 1.00D of prediction error. The I^2^ value was used to test the statistical heterogeneity. A random-effect meta-regression model was used when significant heterogeneity (I^2^ > 50%) were found. Otherwise, a fixed-effect meta-regression model was chosen. Using a Beg funnel plot to test Publication bias. A sensitivity analysis was conducted to assess whether the results were affected by the excessive weight of a single study.

## Supplementary information

Supplementary table and figures.
