# Supplementary material for: Intraocular Lens power calculation after laser refractive surgery: A Meta-Analysis
Source: Sci Rep. 2020 Feb 14;10:2645. doi: 10.1038/s41598-020-59487-1 (PMC7021678; doi:10.1038/s41598-020-59487-1)

Full title:

Intraocular Lens power calculation after laser refractive surgery: A Meta-Analysis

Authors: Hui Chen[1,2,3]*, Xinyi Chen[1,2]*, Hanle Wang[1,2], Zhi Fang[1,2] and Ke Yao[1,2]

Author details

[1] Eye Center of the Second Affiliated Hospital, School of Medicine, Zhejiang University, 310009, Hangzhou, Zhejiang Province, China.

[2] Eye Institute of Zhejiang University, 310009, Hangzhou, Zhejiang Province, China.

[3]The Eye Hospital, Wenzhou Medical University, Wenzhou, Zhejiang, P.R China.

Correspondence should be addressed to: Ke Yao, MD, Professor and Chief, Eye Center of the Second Affiliated Hospital, School of Medicine, Zhejiang University, Eye Institute of Zhejiang University, 310009, Hangzhou, Zhejiang Province, China; Email: xlren@zju.edu.cn.

*These authors contributed equally.

Supplementary Information

Table S1. Newcastle–Ottawa Scale for observational studies.

				NOS scale			
**No.**	**Study ID**	**Country**	**Study design**	**selection**	**comparability**	**outcome**	**total score**
1	Wu 2017	China	prospective cohort	★★★★	★★	★★★	★★★★★★★★★
2	Helaly 2016	Egypt	prospective cohort	★★★	★	★★★	★★★★★★★
3	Huang 2013	America	prospective cohort	★★★	★★	★★★	★★★★★★★★
4	Savini 2010	America	prospective cohort	★★★	★★	★★★	★★★★★★★★
5	Jin 2010	Germany	prospective cohort	★★★★	★★	★★★	★★★★★★★★★
6	Arce 2009	America	prospective cohort	★★★	★	★★★	★★★★★★★
7	Shammas 2007	America	prospective cohort	★★★	★★	★★★	★★★★★★★★
8	Savini 2018	Italy	prospective case series	★★★	★★	★	★★★★★★
9	Savini 2015	America	prospective case series	★★★	★★	★	★★★★★★
10	Wang 2004	America	prospective case series	★★★	★★	★★★	★★★★★★★★

A higher overall score corresponds to a lower risk of bias; a score of six or more (out of nine) indicates a low risk of bias. Each * equals 1 points.

Fig. S1. Forest plots comparing the ME between Haigis-L and Hoffer Q.

	Haigis-L			Hoffer Q				Mean Difference
Study or Subgroup	Mean	SD	Total	Mean	SD	Total	Weight	IV, Fixed, 95% CI
Arce 2009	-0.9	0.82	18	-0.44	0.79	18	52.3%	-0.46 [-0.99, 0.07]
Shammas 2007	-0.22	0.79	15	-0.23	0.75	15	47.7%	0.01 [-0.54, 0.56]
**Total (95% CI)**			**33**			**33**	**100.0%**	**-0.24 [-0.62, 0.14]**

Heterogeneity: Chi² = 1.46, df = 1 (P = 0.23); I² = 32%
Test for overall effect: Z = 1.22 (P = 0.22)

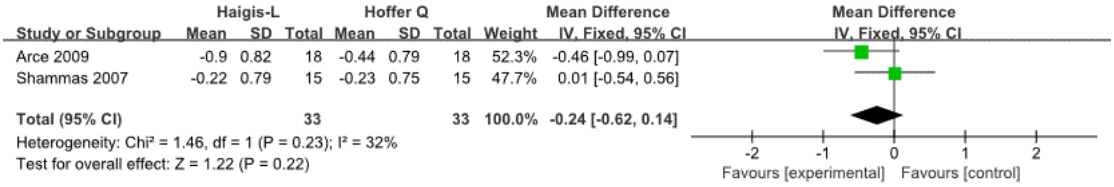

Fig. S2. Forest plots comparing the ME between Haigis-L and Holladay 1.

	Haigis-L			Holladay Ⅰ				Mean Difference
Study or Subgroup	Mean	SD	Total	Mean	SD	Total	Weight	IV, Random, 95% CI
Arce 2009	-0.9	0.82	18	0.15	1.05	18	49.5%	-1.05 [-1.67, -0.43]
Shammas 2007	-0.22	0.79	15	-0.02	0.86	15	50.5%	-0.20 [-0.79, 0.39]
**Total (95% CI)**			**33**			**33**	**100.0%**	**-0.62 [-1.45, 0.21]**

Heterogeneity: Tau² = 0.27; Chi² = 3.81, df = 1 (P = 0.05); I² = 74%
Test for overall effect: Z = 1.46 (P = 0.14)

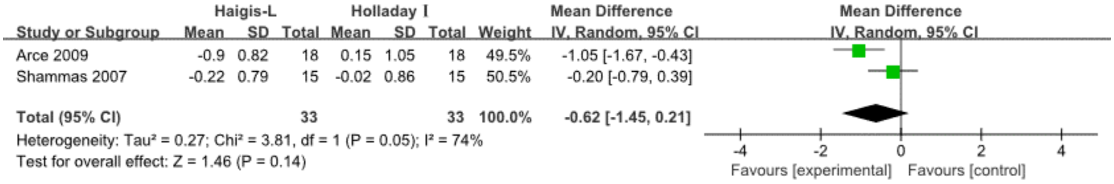

Fig. S3. Forest plots comparing the ME between SRK/T and Holladay1.

	SRK/T			Holladay Ⅰ				Mean Difference
Study or Subgroup	Mean	SD	Total	Mean	SD	Total	Weight	IV, Random, 95% CI
**3.7.1 Single-K**								
Arce 2009	-0.1	0.8	14	-0.14	0.78	14	8.1%	0.04 [-0.55, 0.63]
Arce 2009a	0.32	1.09	18	0.15	1.05	18	6.9%	0.17 [-0.53, 0.87]
Wang 2004	0.87	1.27	11	0.6	1.46	11	3.8%	0.27 [-0.87, 1.41]
Wang 2004a	4.26	3.13	11	4.2	3.61	11	0.8%	0.06 [-2.76, 2.88]
Wang 2004b	0.88	0.81	11	0.63	0.88	11	6.8%	0.25 [-0.46, 0.96]
Wang 2004c	1.94	1	11	1.78	1.01	11	5.7%	0.16 [-0.68, 1.00]
**Subtotal (95% CI)**			**76**			**76**	**32.2%**	**0.15 [-0.18, 0.48]**
Heterogeneity: Tau² = 0.00; Chi² = 0.26, df = 5 (P = 1.00); I² = 0%								
Test for overall effect: Z = 0.90 (P = 0.37)								
**3.7.2 Double-K**								
Savini 2010	1.73	1.23	23	0.74	1.1	23	7.2%	0.99 [0.32, 1.66]
Savini 2010a	1.37	0.83	23	0.53	1	23	8.7%	0.84 [0.31, 1.37]
Savini 2010b	2.18	1.39	5	1.17	1.13	5	2.3%	1.01 [-0.56, 2.58]
Savini 2010c	1.69	0.92	5	0.71	1.05	5	3.5%	0.98 [-0.24, 2.20]
Savini 2015	0.71	1.05	30	0.36	1.2	30	8.3%	0.35 [-0.22, 0.92]
Savini 2018	0.32	0.61	15	-0.01	0.69	15	9.5%	0.33 [-0.14, 0.80]
Shammas 2007	-0.65	0.63	15	-0.02	0.86	15	8.6%	-0.63 [-1.17, -0.09]
Wang 2004	-0.98	0.72	11	-0.62	0.87	11	7.2%	-0.36 [-1.03, 0.31]
Wang 2004a	0.45	0.51	11	0.8	0.74	11	8.7%	-0.35 [-0.88, 0.18]
Wang 2004b	-1.02	1.13	11	-0.67	1.53	11	3.9%	-0.35 [-1.47, 0.77]
**Subtotal (95% CI)**			**149**			**149**	**67.8%**	**0.22 [-0.18, 0.61]**
Heterogeneity: Tau² = 0.27; Chi² = 30.78, df = 9 (P = 0.0003); I² = 71%								
Test for overall effect: Z = 1.07 (P = 0.28)								
**Total (95% CI)**			**225**			**225**	**100.0%**	**0.19 [-0.07, 0.45]**

Heterogeneity: Tau² = 0.14; Chi² = 31.06, df = 15 (P = 0.009); I² = 52%
Test for overall effect: Z = 1.40 (P = 0.16)
Test for subgroup differences: Chi² = 0.06, df = 1 (P = 0.80), I² = 0%

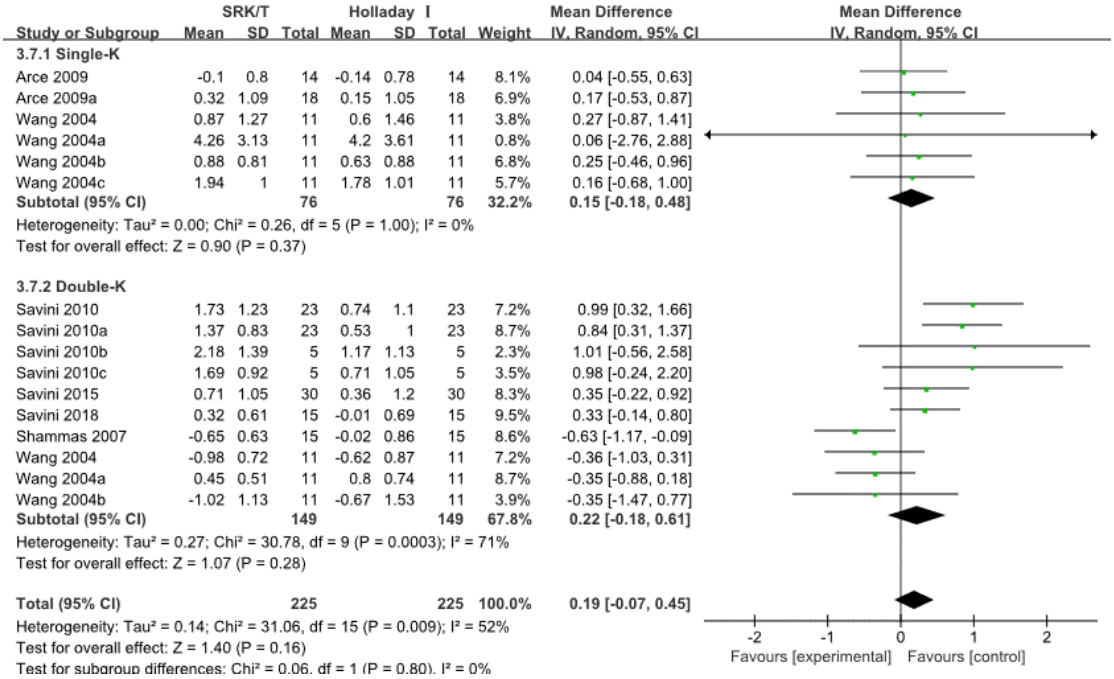

Fig. S4. Forest plots comparing the ME between Holladay 1 and Hoffer Q.

	Holladay I			Hoffer Q				Mean Difference
**Study or Subgroup**	**Mean**	**SD**	**Total**	**Mean**	**SD**	**Total**	**Weight**	**IV, Fixed, 95% CI**
**6.3.1 Single-K**								
Arce 2009	0.9	0.63	18	0.78	0.49	18	20.7%	0.12 [-0.25, 0.49]
Wang 2004	0.8	0.71	11	0.51	0.31	11	13.5%	0.29 [-0.17, 0.75]
Wang 2004a	4.27	3.51	11	3.84	3.29	11	0.3%	0.43 [-2.41, 3.27]
Wang 2004b	1.78	1.01	11	1.25	0.6	11	5.9%	0.53 [-0.16, 1.22]
Wang 2004c	1.25	0.9	11	0.83	0.93	11	4.8%	0.42 [-0.34, 1.18]
**Subtotal (95% CI)**			**62**			**62**	**45.2%**	**0.26 [0.01, 0.51]**
Heterogeneity: Chi² = 1.33, df = 4 (P = 0.86); I² = 0%								
Test for overall effect: Z = 2.02 (P = 0.04)								
**6.3.2 Double-K**								
Shammas 2007	0.64	0.55	15	0.68	0.33	15	26.8%	-0.04 [-0.36, 0.28]
Wang 2004	0.84	0.63	11	0.69	0.51	11	12.3%	0.15 [-0.33, 0.63]
Wang 2004a	1.24	1.08	11	1.08	0.93	11	4.0%	0.16 [-0.68, 1.00]
Wang 2004b	0.85	0.67	11	0.88	0.49	11	11.7%	-0.03 [-0.52, 0.46]
**Subtotal (95% CI)**			**48**			**48**	**54.8%**	**0.02 [-0.21, 0.25]**
Heterogeneity: Chi² = 0.56, df = 3 (P = 0.91); I² = 0%								
Test for overall effect: Z = 0.17 (P = 0.87)								
**Total (95% CI)**			**110**			**110**	**100.0%**	**0.13 [-0.04, 0.30]**
Heterogeneity: Chi² = 3.82, df = 8 (P = 0.87); I² = 0%								
Test for overall effect: Z = 1.49 (P = 0.14)								
Test for subgroup differences: Chi² = 1.92, df = 1 (P = 0.17), I² = 48.0%								

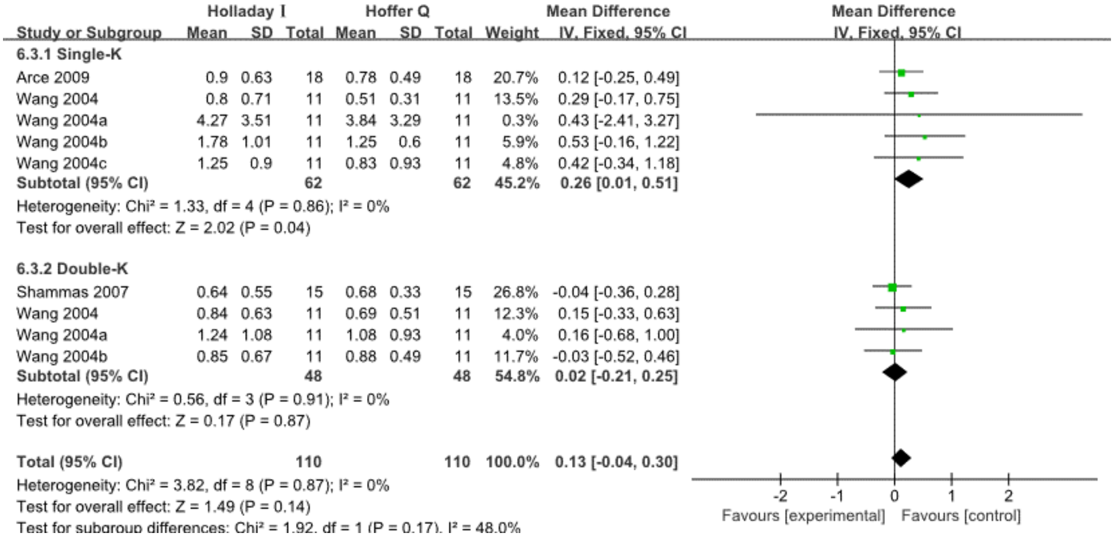

Supplement: Supplementary file 1 — Supplementary table and figures. [file 41598_2020_59487_MOESM1_ESM.pdf]
